# Association of AI-derived abdominal organ volumetry with postoperative outcomes in clear cell renal cell carcinoma: a multicentre study

**DOI:** 10.1080/07853890.2026.2730502

**Published:** 2026-09-10

**Authors:** Jie Lou, Yi Ren, Bingxin Gong, Yusheng Guo, Dingyang Lv, Shuai Shan, Qiang Lu, Aoxin Sun, Jijun Wu, Yuhang Chen, Xin Yao, Lian Yang

**Affiliations:** a Department of Radiology, Union Hospital, Tongji Medical College, Huazhong University of Science and Technology, Wuhan, China; b Hubei Provincial Clinical Research Center for Precision Radiology & Interventional Medicine, Wuhan, China; c Hubei Key Laboratory of Molecular Imaging, Wuhan, China; d First Clinical Medical College of Shanxi Medical University, Taiyuan, Shanxi, China; e Department of Urology, First Hospital of Shanxi Medical University, Taiyuan, Shanxi, China; f Department of Radiology, The First Affiliated Hospital of Nanjing Medical University, Nanjing, Jiangsu Province, China; g Department of Urology, The First Affiliated Hospital of Nanjing Medical University, Nanjing, Jiangsu Province, China; h Department of Genitourinary Oncology, Tianjin Medical University Cancer Institute and Hospital, National Clinical Research Center for Cancer, Key Laboratory of Cancer Prevention and Therapy, Tianjin’s Clinical Research Center for Cancer, Tianjin, China

**Keywords:** Clear cell renal cell carcinoma, nephrectomy, computed tomography, abdominal organ volume, prognosis, artificial intelligence

## Abstract

**Background:**

CT-derived body composition parameters are associated with clinical outcomes in clear cell renal cell carcinoma (ccRCC), but the prognostic value of abdominal solid organ volumes remains unclear.

**Methods:**

In this multicentre study, 1,720 patients with ccRCC who underwent nephrectomy across four centres and 3,052 healthy controls were included. A deep learning algorithm was used to automatically quantify abdominal organ volumes. Organ volume indices were calculated by normalizing volumes to body surface area. Associations between abdominal organ volumes and recurrence-free survival (RFS) and overall survival (OS) were evaluated using centre-stratified Cox regression models. Multivariable models were adjusted for age, gender, tumour size, laterality, surgical approach, pT stage, pN stage, International Society of Urological Pathology grade and tumor necrosis.

**Results:**

During a median follow-up of 51.6 (49.2–52.8) months, 165 patients experienced recurrence and 95 died. Organ volume distributions were similar across centres. Compared with healthy controls, ccRCC patients showed higher spleen and adrenal gland volume indices and lower pancreatic volume index. In multivariable Cox analyses, higher liver volume (HR = 1.42, *P* adj = 0.035), liver volume index (HR = 1.31, *P* adj = 0.035), right adrenal gland volume (HR = 1.34, *P* adj = 0.035) and right adrenal gland volume index (HR = 1.29, *P* adj = 0.035) were independently associated with worse OS. No parameter was associated with RFS after multiple-testing correction.

**Conclusions:**

Higher liver and right adrenal gland volumes were associated with worse OS in ccRCC. These findings warrant external validation and further mechanistic investigation.

## Introduction

More than 434,419 new cases of renal cell carcinoma were diagnosed worldwide in 2022 [1], of which the most prevalent subtype is clear cell renal cell carcinoma (ccRCC), accounting for approximately 70–80% of all cases [2,3]. ccRCC is characterized by significant molecular and immune heterogeneity, which plays crucial roles in tumour progression and patient outcomes [4]. Although surgical resection remains the primary approach for the treatment of localized ccRCC, 20–30% of patients would experience recurrence or metastasis, which affects the survival rate of patients [5,6]. Therefore, there is an urgent need for novel noninvasive biomarkers to predict the prognostic risk of ccRCC.

In recent years, body composition analysis derived from routine computed tomography (CT) imaging has emerged as a valuable tool in oncology research. Accumulating evidence has shown that skeletal muscle mass and abdominal fat distribution are associated with survival outcomes in patients with various malignancies including ccRCC [7,8]. Bradshaw et al. analysed data from 1,022 patients with ccRCC and found that poor OS was associated with lower skeletal muscle radiodensity and greater visceral adipose tissue radiodensity [9]. These findings suggest that CT-derived body composition parameters may reflect systemic metabolic and inflammatory states that influence tumour progression and patient outcomes.

However, most existing studies have primarily focused on muscle and adipose tissue, while the prognostic potential of abdominal solid organ morphology has been largely overlooked. The changes of organ volumes may reflect metabolic disturbances and health status of the body. For example, adrenal gland hypertrophy may reflect hypothalamic-pituitary-adrenal (HPA) axis activation [10]. In addition, Bae et al. reported that spleen volume divided by body surface area (BSA) was a significant influencing factor for overall survival (OS) after hepatic resection in patients with hepatocellular carcinoma [11]. A study in pancreatic ductal adenocarcinoma demonstrated that a lower BSA-adjusted pancreatic volume (< 40 mL/m^2^) was independently associated with markedly poorer survival [12]. These findings suggest that abdominal organ volumes may serve as imaging biomarkers reflecting the systemic physiological and immunometabolic status of cancer patients. However, whether abdominal organ volumes have prognostic value for patients with ccRCC remains unknown. Moreover, manual segmentation of abdominal organs is labour-intensive and highly time consuming, which is impractical for large-scale clinical studies [13]. Advances in deep learning-based methods for image analysis, particularly fully automated segmentation frameworks such as nnU-Net [14], provide a new possibility for accurate quantification of organ volumes from routine CT scans.

Therefore, in this multicentre study, we leveraged a deep learning-based algorithm to automatically quantify the volumes of five major abdominal organs including the liver, spleen, pancreas and bilateral adrenal glands from routine preoperative CT scans. We further compared organ size between patients with ccRCC and healthy individuals and investigated whether these volumetric parameters and their normalized indices were associated with postoperative outcomes. RFS and OS were considered primary endpoints, and the five organ volumes and their BSA-normalized indices were assessed in parallel as measures of abdominal organ size. This study aims to provide new insights into the prognostic value of abdominal organ volumes and to explore its potential utility in improving preoperative risk stratification for patients with ccRCC.

## Materials and methods

### Patient cohorts and study design

**Figure 1.**
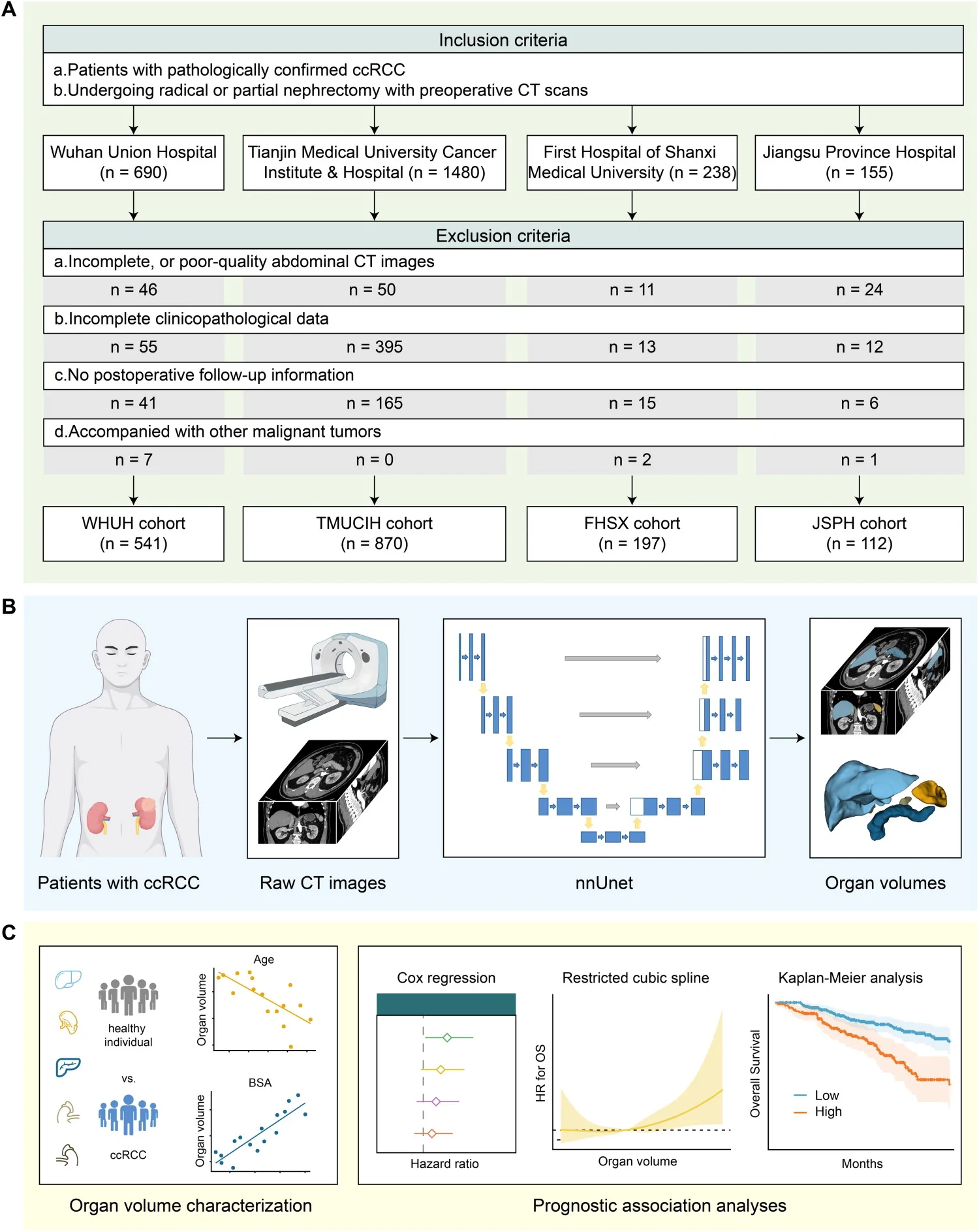
The overall design of this study. (A) Flowchart of patient inclusion and exclusion. (B) Deep learning-based segmentation and quantification of abdominal organ volumes. (C) The associations between organ volumes and clinical outcomes. ccRCC, clear cell renal cell carcinoma; CT, computed tomography; WHUH, Wuhan Union Hospital; TMUCIH, Tianjin Medical University Cancer Institute & Hospital; FHSX, First Hospital of Shanxi Medical University; JSPH, Jiangsu Province Hospital.

In this study, we retrospectively collected multimodal data from ccRCC patients who underwent surgery across four centres in China: Wuhan Union Hospital (WHUH, January 2015 to September 2023), Tianjin Medical University Cancer Institute & Hospital (TMUCIH, January 2017 to December 2023), First Hospital of Shanxi Medical University (FHSX, May 2018 to December 2021) and Jiangsu Province Hospital (JSPH, January 2019 to December 2021). As shown in Figure 1, all the study participants met the following inclusion criteria: (1) pathologically confirmed diagnosis of ccRCC and (2) patients received radical or partial nephrectomy with preoperative CT scans. Exclusion criteria included incomplete or poor-quality abdominal CT scans, missing clinicopathological information, loss to follow-up and the presence of diseases that affect abdominal organ volumes. Healthy controls were adults undergoing routine health examinations at WHUH. The inclusion criteria were age over 18 years, availability of abdominal CT imaging and no known malignancy or major systemic disease based on medical history, clinical records and laboratory examinations. Exclusion criteria were: (1) incomplete CT scans or poor image quality and (2) missing clinical information such as height and weight. This study complies with the Declaration of Helsinki and was approved by the ethics committee of WHUH, TMUCIH, FHSX and JSPH.

The overall design of this study is shown in Figure 1. We collected abdominal CT images from all enrolled patients and used a deep learning algorithm to automatically segment and quantify the volumes of five abdominal organs including the spleen, liver, pancreas and the left and right adrenal glands. Each organ index was subsequently calculated by normalizing the organ volume to the patient’s BSA. We then performed correlation analyses to examine the relationships among abdominal organ volumes and between organ volumes and clinical variables. In addition, Cox regression analyses were conducted to evaluate the associations between abdominal organ metrics and the prognosis of patients with ccRCC.

### Clinical and prognostic data

Clinical data including age, gender, height, weight, tumour laterality, date of surgery, surgical approach, past history and pathological information were extracted from electronic medical records of the four medical centres. BSA was calculated according to the Mosteller formula [15]. Follow-up data were obtained from outpatient visits or telephone interviews. The primary endpoints were OS and recurrence-free survival (RFS). OS was defined as the time from surgery to death or the last follow-up. RFS was defined as the time from surgery to tumour recurrence or the last follow-up.

### Image acquisition and preprocessing

Preoperative contrast-enhanced CT examinations performed within 4 weeks before surgery were collected from all participating institutions. Corticomedullary-phase images were extracted from the multiphase contrast-enhanced CT series for volumetric analysis. Detailed scanner and acquisition parameters are provided in Table S1. To minimize inter-centre variability, all images underwent a standardized preprocessing pipeline. DICOM images were anonymized and converted to NIfTI format, resampled to an isotropic voxel spacing of 1 × 1 × 1 mm³ using trilinear interpolation, and intensity-normalized using a fixed abdominal window (window width, 350 HU; window level, 50 HU).

### Abdominal organ volume measurements

Automated organ segmentation was performed using an internally trained nnU-Net-based (https://github.com/MIC-DKFZ/nnUNet) model implemented within the TotalSegmentator framework (https://github.com/wasserth/TotalSegmentator) [14,16]. After generating the segmentation mask for each organ, volumes were calculated by multiplying the number of voxels within each organ by the volume of a voxel. To account for individual variation in organ size, organ volumes were normalized by BSA to derive the corresponding organ indices. Because the volumes of hollow abdominal organs such as the stomach and gallbladder are highly dependent on their filling state, and renal tumours may alter kidney size, this study focused on measuring the volumes of solid organs, including the spleen, liver, pancreas and bilateral adrenal glands. Therefore, we obtained the volumes and corresponding indices for five solid organs, with detailed definitions and measurement methods provided in Table S2. To evaluate the accuracy of the automated segmentation, a validation subset comprising 20 randomly selected patients from each participating centre was established. Manual segmentation of the liver, spleen, pancreas and bilateral adrenal glands was independently performed by an experienced radiologist who was blinded to the patients’ clinical information and outcomes and used as the reference standard. The automated and manual segmentation results were subsequently compared using the Dice similarity coefficient (DSC), intraclass correlation coefficient (ICC) and Bland-Altman analysis.

### Statistical analyses

Continuous variables are expressed in medians and interquartile ranges (IQR), and categorical variables are expressed in frequencies and percentages. Continuous variables were compared using t-tests or Wilcoxon rank-sum tests. Propensity score matching (PSM) was performed based on age and gender using nearest-neighbour matching with a calliper of 0.2 SD of the propensity score. Balance before and after matching was assessed using standardized mean differences (SMDs). After matching, BSA-normalized organ volume indices were compared using paired Wilcoxon signed-rank tests to account for the matched-pair design. Spearman’s rank correlations were used to assess the relationships among organ volumes and between organ volumes and clinical variables. Univariable and multivariable Cox proportional hazards models stratified by centre were used to evaluate the association between abdominal organ parameters and prognosis. Hazard ratios (HR) are expressed per 1 standard deviation increase for organ volumes and indices. For all Cox proportional hazards analyses, the proportional hazards assumption was met. To account for multiple testing across organ measures, *P* values were adjusted using the Benjamini-Hochberg false discovery rate (FDR) method, with FDR-adjusted *P* (*P* adj) < 0.05 considered statistically significant. Restricted cubic spline analyses were performed to assess the nonlinear associations with OS, using the same covariate adjustment and centre stratification as the multivariable Cox models. The optimal cut-off values for organ volumes and indices were determined using the surv_cutpoint function in the R package survminer. Survival analyses were performed using the Kaplan-Meier method, and the difference between curves was assessed by log-rank test. The restricted mean survival time (RMST) and RMST difference were calculated using the R package survRM2. To evaluate the incremental prognostic value of organ volume measures, pTNM stage and the Leibovich score were used as reference clinical risk models. Model discrimination was assessed using Harrell’s C-index, and incremental value was evaluated using the net reclassification improvement (NRI) and integrated discrimination improvement (IDI). All statistical tests were two-sided and *P*-value less than 0.05 was considered statistically significant. All analyses were performed using the statistical software package R version 4.4.1.

## Results

### Patient baseline characteristics

As shown in Figure 1A, a total of 1,720 patients with pathologically confirmed ccRCC from four medical centres in China were ultimately included in this study after applying the predefined inclusion and exclusion criteria. During a median follow-up of 51.6 (49.2–52.8) months, 165 patients experienced recurrence and 95 died. The demographic and baseline clinical characteristics of the enrolled patients are detailed in Table 1. The median age across the four cohorts ranged from 56.0 to 58.0 years, and the male patients accounted for the majority of cases, with proportions ranging from 64.8% to 67.9%. Although there were differences in some characteristics among the four cohorts, these variations reflect real-world clinical heterogeneity and enhance the representativeness of the study population. In addition to the ccRCC cohort, a large healthy adult control cohort (*n* = 3,052) was included to compare abdominal organ size between patients with ccRCC and healthy individuals.

**Table 1.** Patient demographics and baseline characteristics.

Characteristics	WHUH cohort (*n* = 541)	TMUCIH cohort (*n* = 870)	FHSX cohort (*n* = 197)	JSPH cohort (*n* = 112)
Age	56.0 (49.0, 64.0)	58.0 (52.0, 65.0)	57.0 (51.0, 65.0)	56.5 (49.0, 65.0)
BSA	1.78 (1.63, 1.90)	1.86 (1.71, 2.01)	1.82 (1.68, 1.93)	1.78 (1.69, 1.88)
Gender				
Male	354 (65.4%)	564 (64.8%)	128 (65.0%)	76 (67.9%)
Female	187 (34.6%)	306 (35.2%)	69 (35.0%)	36 (32.1%)
Tumour size ≥ 10 cm				
No	516 (95.4%)	848 (97.5%)	191 (97.0%)	108 (96.4%)
Yes	25 (4.6%)	22 (2.5%)	6 (3.0%)	4 (3.6%)
Laterality				
Left	275 (50.8%)	434 (49.9%)	92 (46.7%)	52 (46.4%)
Right	266 (49.2%)	436 (50.1%)	105 (53.3%)	60 (53.6%)
Surgical approach				
PN	296 (54.7%)	321 (36.9%)	63 (32.0%)	60 (53.6%)
RN	245 (45.3%)	549 (63.1%)	134 (68.0%)	52 (46.4%)
pT stage				
T1	430 (79.5%)	677 (77.8%)	173 (87.8%)	97 (86.6%)
T2	35 (6.5%)	84 (9.7%)	20 (10.2%)	5 (4.5%)
T3	76 (14.0%)	109 (12.5%)	4 (2.0%)	10 (8.9%)
pN stage				
N0/x	534 (98.7%)	866 (99.5%)	197 (100.0%)	112 (100.0%)
N1	7 (1.3%)	4 (0.5%)	0 (0.0%)	0 (0.0%)
Hypertension				
Yes	184 (34.0%)	134 (15.4%)	88 (44.7%)	35 (31.3%)
No	357 (66.0%)	235 (27.0%)	109 (55.3%)	77 (68.7%)
Missing	0 (0.0%)	501 (57.6%)	0 (0.0%)	0 (0.0%)
Diabetes				
Yes	72 (13.3%)	62 (7.1%)	24 (12.2%)	18 (16.1%)
No	469 (86.7%)	307 (35.3%)	173 (87.8%)	94 (83.9%)
Missing	0 (0.0%)	501 (57.6%)	0 (0.0%)	0 (0.0%)
Smoking				
Yes	142 (26.2%)	130 (14.9%)	45 (22.8%)	17 (15.2%)
No	399 (73.8%)	239 (27.5%)	152 (77.2%)	95 (84.8%)
Missing	0 (0.0%)	501 (57.6%)	0 (0.0%)	0 (0.0%)
ISUP grade				
I	47 (8.7%)	57 (6.6%)	25 (12.7%)	13 (11.6%)
II	306 (56.6%)	547 (62.8%)	116 (58.9%)	60 (53.6%)
III	164 (30.3%)	229 (26.3%)	51 (25.9%)	33 (29.5%)
IV	24 (4.4%)	37 (4.3%)	5 (2.5%)	6 (5.3%)
Necrosis				
No	456 (84.3%)	770 (88.5%)	160 (81.2%)	106 (94.6%)
Yes	85 (15.7%)	100 (11.5%)	37 (18.8%)	6 (5.4%)
Leibovich group				
Low	361 (66.7%)	568 (65.3%)	136 (69.0%)	85 (75.9%)
Intermediate	138 (25.5%)	239 (27.5%)	57 (28.9%)	20 (17.9%)
High	42 (7.8%)	63 (7.2%)	4 (2.0%)	7 (6.3%)
Recurrence	73 (13.5%)	61 (7.0%)	16 (8.1%)	15 (13.4%)
Deaths	22 (4.1%)	53 (6.1%)	13 (6.6%)	7 (6.2%)
Median follow-up	41.5 (39.0, 44.8)	52.1 (49.4, 54.7)	55.0 (53.0, 59.0)	53.7 (51.0, 56.4)

Note: WHUH, Wuhan Union Hospital; TMUCIH, Tianjin Medical University Cancer Institute & Hospital; FHSX, First Hospital of Shanxi Medical University; JSPH, Jiangsu Province Hospital; BSA, body surface area; PN, partial nephrectomy; RN, radical nephrectomy; ISUP, International Society of Urological Pathology.

### Preoperative abdominal organ size in patients with ccRCC

**Figure 2.**
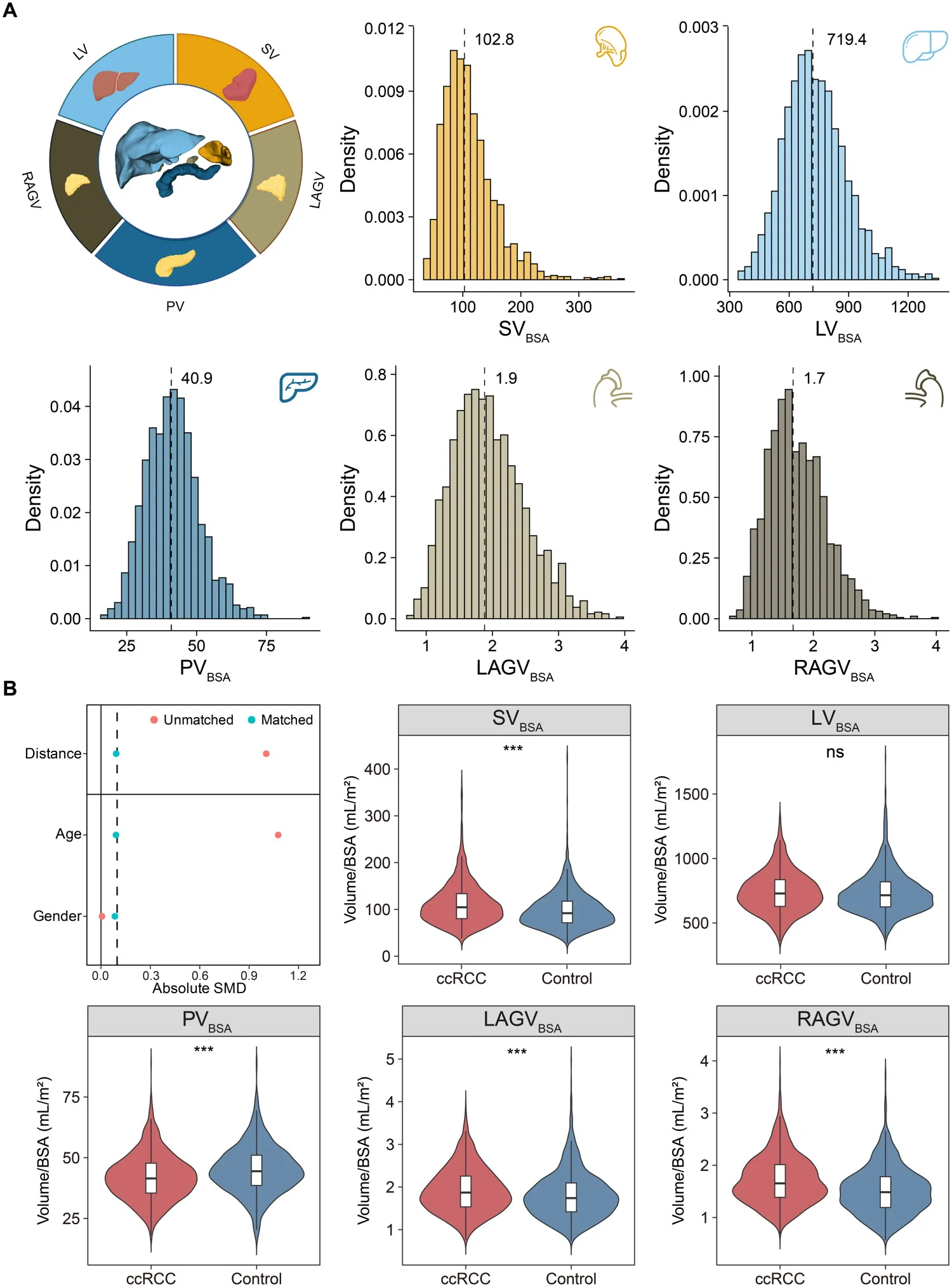
(A) The distributions of the five organ volume indices. (B) Comparison of organ volume indices between patients with ccRCC and healthy individuals. BSA, body surface area; SV, spleen volume; LV, liver volume; PV, pancreas volume; LAGV, left adrenal gland volume; RAGV, right adrenal gland volume; ccRCC, clear cell renal cell carcinoma.

In this study, we used the deep learning algorithm to automatically measure the spleen volume (SV), liver volume (LV), pancreas volume (PV), left adrenal gland volume (LAGV) and right adrenal gland volume (RAGV) from preoperative CT scans. The overall distributions of these organ volumes are presented in Figure S1. Taking into account differences in body size, volume indices were calculated by normalizing each organ volume to the BSA, yielding the corresponding organ volume indices: SV_BSA_, LV_BSA_, PV_BSA_, LAGV_BSA_ and RAGV_BSA_. Figure 2A shows the distributions of the five organ volume indices. The distributions of abdominal organ volumes and their indices were similar across the participating hospitals (Figure S2). The automated segmentation showed good agreement with manual segmentation across all five organs (Table S3). Dice coefficients ranged from 0.848 to 0.966, with ICCs ranging from 0.938 to 0.987. Bland-Altman analyses further supported the agreement between automated and manual volume measurements (Figure S3).

**Figure 3.**
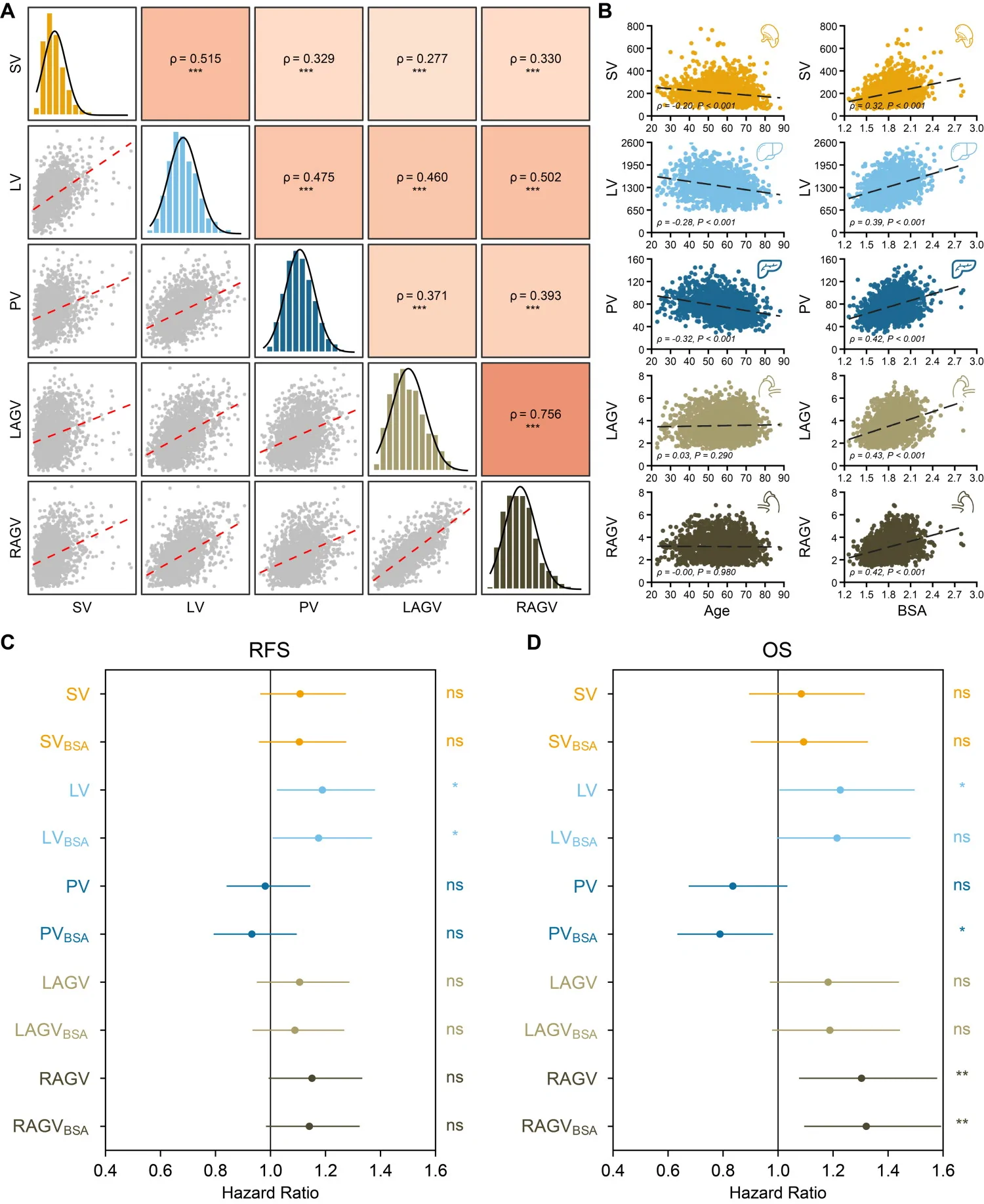
(A) Correlation matrix of abdominal organ volumes. (B) Scatter plots of the relationships between abdominal organ volumes and age and BSA. (C) Associations between abdominal organ volumes and RFS in ccRCC. (D) Associations between abdominal organ volumes and OS in patients with ccRCC. Hazard ratios are expressed per 1 standard deviation increase for organ volumes and indices. * represented *p* < 0.05, ** represented *p* < 0.01, *** represented *p* < 0.001, ns represented *p* > 0.05. BSA, body surface area; SV, spleen volume; LV, liver volume; PV, pancreas volume; LAGV, left adrenal gland volume; RAGV, right adrenal gland volume; RFS, recurrence-free survival; OS, overall survival.

Table S4 outlines the demographic characteristics of the ccRCC and healthy control cohorts before and after PSM. Matching was performed based on age and gender to minimize baseline differences between the two groups. After PSM, the demographic characteristics were well balanced, with all SMDs under 0.10 (Figure 2B). Compared with the healthy control group, patients with ccRCC had significantly higher SV_BSA_, LAGV_BSA_, and RAGV_BSA_ and lower PV_BSA_ (all *p* < 0.001). LV_BSA_ was not significantly different between the two groups (Figure 2B). To minimize the potential centre-related confounding, we repeated the comparison using only patients with ccRCC and healthy controls from WHUH. After propensity score matching, 540 matched pairs were obtained (Table S5). SV_BSA_, LAGV_BSA_ and RAGV_BSA_ remained significantly higher in patients with ccRCC, whereas no significant differences were observed in LV_BSA_ or PV_BSA_ (Figure S4).

In addition, correlation analyses revealed varying degrees of positive associations among abdominal organ volumes, with Spearman’s rank correlations ranging from 0.277 to 0.756 (all *p* < 0.001). The strongest correlation was observed between bilateral adrenal gland volumes (ρ = 0.756, *p* < 0.001) (Figure 3A). As shown in Figure 3B, SV, LV and PV were negatively correlated with age (ρ= −0.20, ρ= −0.28 and ρ= −0.32, respectively), whereas all abdominal organ volumes were positively correlated with BSA (ρ ranging from 0.32 to 0.43, all *p* < 0.001).

### Association between abdominal organ volumes and postoperative outcomes

We next investigated the prognostic relevance of abdominal organ volumes for postoperative recurrence and survival in patients with ccRCC. In the univariable Cox regression analyses, we found that LV (HR = 1.19, *p* = 0.023) and LV_BSA_ (HR = 1.18, *p* = 0.039) were potential risk factors for RFS in patients with ccRCC (Figure 3C, Table S6). However, no organ volume measure remained significantly associated with RFS after FDR correction. Higher RAGV (HR = 1.30, *P* adj = 0.033), and RAGV_BSA_ (HR = 1.32, *P* adj = 0.033) were significantly associated with worse OS after FDR correction (Figure 3D, Table 2).

**Table 2.** Associations between abdominal organ volumes and overall survival in ccRCC patients.

Variables	Univariate			Multivariate		
	HR (95% CI)	*P* value	*P* adj	HR (95% CI)*	*P* value	*P* adj
Spleen						
SV	1.08 (0.89, 1.32)	0.410	0.410	1.05 (0.84, 1.30)	0.680	0.753
SV_BSA_	1.09 (0.90, 1.33)	0.368	0.409	1.03 (0.84, 1.27)	0.753	0.753
Liver						
LV	1.23 (1.01, 1.50)	**0.045**	0.110	1.42 (1.12, 1.79)	**0.003**	**0.035**
LV_BSA_	1.21 (1.00, 1.48)	0.055	0.110	1.31 (1.06, 1.62)	**0.014**	**0.035**
Pancreas						
PV	0.84 (0.68, 1.03)	0.099	0.123	0.95 (0.74, 1.23)	0.697	0.753
PV_BSA_	0.79 (0.63, 0.98)	**0.033**	0.110	0.90 (0.72, 1.14)	0.385	0.550
Left adrenal gland						
LAGV	1.18 (0.97, 1.44)	0.097	0.123	1.19 (0.95, 1.49)	0.121	0.243
LAGV_BSA_	1.19 (0.98, 1.44)	0.082	0.123	1.15 (0.94, 1.41)	0.182	0.303
Right adrenal gland						
RAGV	1.30 (1.08, 1.58)	**0.007**	**0.033**	1.34 (1.08, 1.67)	**0.008**	**0.035**
RAGV_BSA_	1.32 (1.10, 1.59)	**0.004**	**0.033**	1.29 (1.05, 1.57)	**0.013**	**0.035**

Note: ccRCC, clear cell renal cell carcinoma; BSA, body surface area; HR, hazard ratio; CI, confidence interval; SV, spleen volume; LV, liver volume; PV, pancreas volume; LAGV, left adrenal gland volume; RAGV, right adrenal gland volume; ISUP, International Society of Urological Pathology.

Hazard ratios are expressed per 1 standard deviation increase for continuous variables.

* Adjusted for age, gender, tumour size, laterality, surgical approach, pT stage, pN stage, ISUP grade, and tumour necrosis, with the baseline hazard stratified by centre.

**Figure 4.**
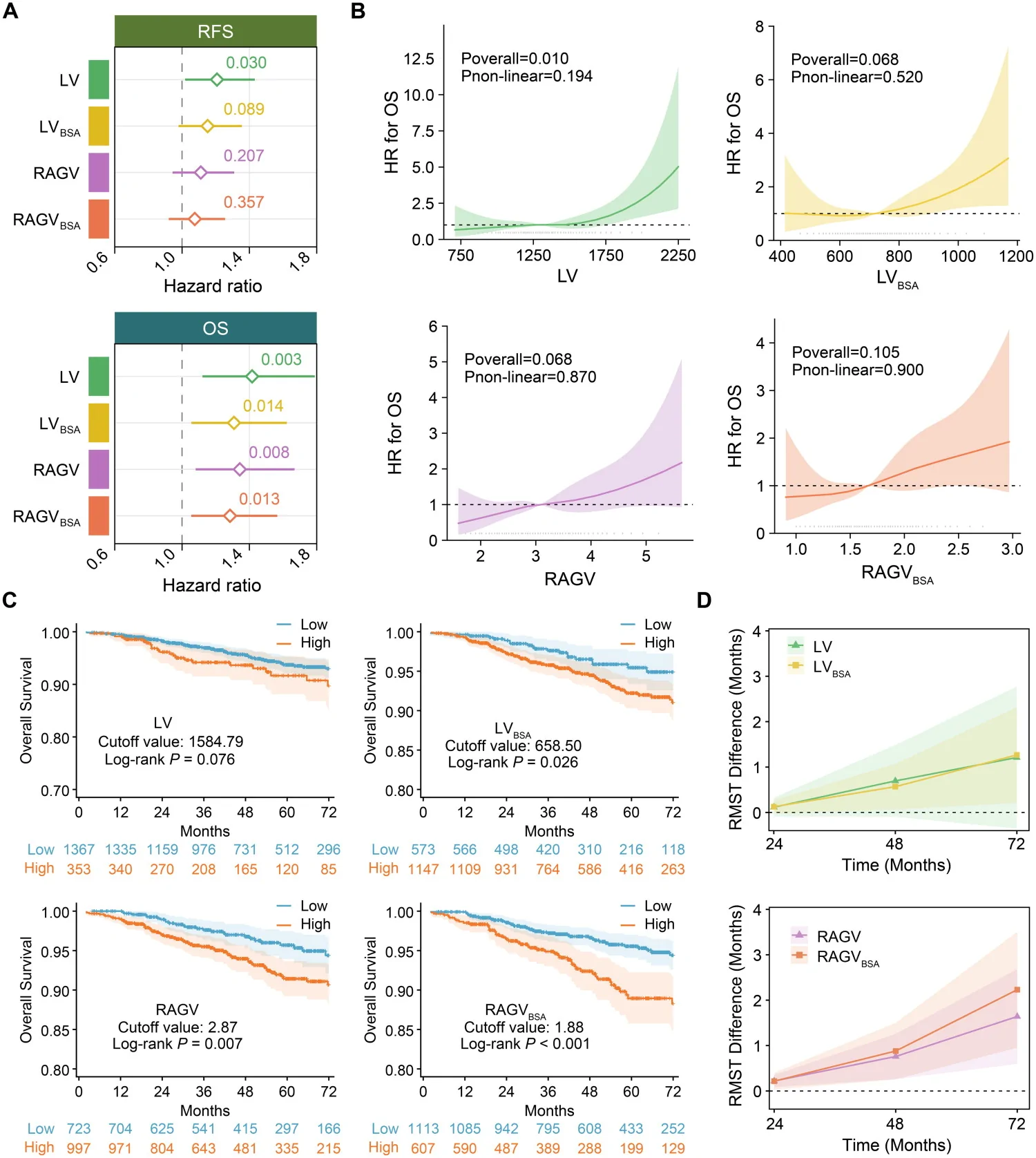
(A) Multivariable centre-stratified Cox regression analyses of organ volume metrics for RFS and OS. (B) Multivariable centre-stratified restricted cubic spline analyses of LV, LVBSA, RAGV and RAGVBSA for OS. (C) Kaplan-Meier curves for OS according to low and high organ volume groups. Survival curves were displayed up to 72 months, when >10% of patients remained at risk. (D) RMST differences between the low and high groups at 24, 48 and 72 months. Hazard ratios are expressed per 1-SD increase in organ volume metrics. RFS, recurrence-free survival; OS, overall survival; BSA, body surface area; LV, liver volume; RAGV, right adrenal gland volume. HR, hazard ratio; RMST, restricted mean survival time; SD, standard deviation.

To account for potential confounding, multivariable Cox regression analyses were performed after adjustment for age, gender, tumour size, laterality, surgical approach, pT stage, pN stage, ISUP grade and tumour necrosis. After adjustment for clinical covariates, higher LV (HR = 1.42, *P* adj = 0.035), LV_BSA_ (HR = 1.31, *P* adj = 0.035), RAGV (HR = 1.34, *P* adj = 0.035) and RAGV_BSA_ (HR = 1.29, *P* adj = 0.035) remained independently associated with worse OS (Figure 4, Table 2). Centre-specific subgroup analyses showed no evidence of significant heterogeneity across institutions for LV, LV_BSA_, RAGV or RAGV_BSA_ (all *P* for interaction > 0.05; Figure S**5**). In contrast, organ volumes and indices did not show statistical significance as prognostic factors for RFS (Figure 4, Table S6). To account for death before recurrence as a competing event, we further performed Fine-Gray competing-risk regression, which yielded similar results (Table S7). Sensitivity analyses evaluating raw organ volumes with BSA included as a separate covariate yielded results consistent with the primary analyses (Table S8).

Restricted cubic spline analyses showed no evidence of significant nonlinear associations between LV, LV_BSA_, RAGV, or RAGV_BSA_ and OS (all *P* for nonlinearity > 0.05, Figure 4B). The incremental prognostic value of organ volume measures was further evaluated against established clinical risk models. Adding RAGV or RAGV_BSA_ to pTNM stage significantly improved the C-index, NRI and IDI (all *p* < 0.05). When added to the Leibovich score, RAGV and RAGV_BSA_ significantly improved NRI and IDI, although the increases in C-index did not reach statistical significance (Table S**9**).

Furthermore, we performed Kaplan-Meier survival analysis to examine the impact of LV, LV_BSA_, RAGV and RAGV_BSA_ on the survival of patients with ccRCC. The results of the Kaplan-Meier analysis are presented in Figure 4C. Patients with higher LV_BSA_, RAGV or RAGV_BSA_ had significantly poorer OS compared to the low groups (log-rank *p* < 0.05). Similar results were obtained in the RMST analyses, further supporting the adverse prognostic impact of higher LV_BSA_, RAGV and RAGV_BSA_ (Figure 4D; Table S10).

## Discussion

In this multicentre study, we characterized the preoperative abdominal organ volumes of patients with ccRCC and investigated whether these organ volumes and their normalized indices were associated with postoperative outcomes in ccRCC. This study integrated large-scale clinical and imaging data from four medical institutions in China and revealed several important findings. First, patients with ccRCC exhibited distinct abdominal organ size patterns compared with healthy individuals, especially in terms of enlarged spleen and adrenal glands and decreased pancreatic volume. Second, among multiple abdominal organs, liver and right adrenal gland volumes, as well as their corresponding indices, were significantly associated with OS, indicating that preoperative abdominal organ size may represent a previously underrecognized biomarker of cancer prognosis.

We observed that patients with ccRCC had larger spleen and adrenal gland volumes and smaller pancreatic volumes compared with healthy individuals. These differences may reflect broader systemic alterations associated with cancer; however, the underlying mechanisms remain uncertain. One possible hypothesis is that cancer-associated immune alterations may contribute to myeloid-derived suppressor cell (MDSC) accumulation in the spleen and potentially influence splenic volume [17–19]. Similarly, the observed adrenal enlargement might be related to cancer-associated proinflammatory signalling and chronic stress responses involving the HPA axis [20–22]. Reduced pancreatic volume may also be associated with cancer-related metabolic alterations, including dysregulation of lipid metabolism [23,24]. However, the precise mechanisms through which these changes are regulated have remained unclear and further studies are warranted to elucidate the underlying mechanisms.

To the best of our knowledge, this is the first study showing that abdominal organ volumes are linked to postoperative outcomes in patients with ccRCC. A study among patients with metastatic renal cell carcinoma found that baseline splenic volume was not significantly associated with the prognosis of patients receiving immunotherapy, which is consistent with our findings [25]. We observed that larger LV and LV_BSA_ were associated with poorer OS. One possible explanation is that increased liver volume may reflect underlying metabolic alterations, including hepatic steatosis and systemic inflammation [26–28]. Previous studies have demonstrated that these abnormalities are also independently associated with the progression of renal cell carcinoma [29]. Nevertheless, this hypothesis remains speculative and warrants further investigation. Moreover, our study found that right adrenal gland volume was also associated with postoperative outcomes in patients with ccRCC. Adrenal enlargement may reflect chronic activation of the HPA axis and endocrine dysregulation [10,21]. Askani et al. reported that adrenal gland volume may serve as an indirect marker of impaired glucose metabolism and dysfunction of the HPA axis [30]. Interestingly, this association was observed only for the right adrenal gland but not the left. Although anatomical and functional asymmetries between the bilateral adrenal glands have been reported [31], the biological basis underlying this finding remains unclear.

This study employed a deep learning-based automated algorithm to perform organ segmentation and quantitative analysis of abdominal CT images in patients with ccRCC. Similar to previous studies, we observed varying degrees of positive correlations among abdominal organ volumes, and organ size was positively associated with BSA [32,33], reflecting inherent anatomical and physiological relationships. In addition, the volumes of the spleen, liver and pancreas gradually decreased with increasing age, consistent with the age-related organ atrophy processes [34]. In recent years, accumulating evidence suggests that CT-derived body composition metrics can predict clinical outcomes in patients with ccRCC. However, previous studies have largely focused on skeletal muscle and adipose tissue [35,36]. Our study extends this paradigm to abdominal organ volumes, suggesting that they may serve as overlooked CT-derived biomarkers of systemic physiological states relevant to long-term survival. Notably, the observed associations were specific to OS, whereas no organ volume parameter remained significantly associated with RFS after multivariable adjustment. This pattern suggests that abdominal organ volumes may primarily reflect the patient’s overall physiological condition rather than tumour-specific biological aggressiveness. Importantly, this approach does not require additional scans, radiation exposure or patient burden. A single preoperative CT scan can simultaneously evaluate tumour characteristics, quantify multiple abdominal organ volumes and provide survival risk stratification.

Despite these strengths, several limitations should be acknowledged. First, the retrospective design may have introduced selection bias and residual confounding. Several potentially relevant variables, including comorbidities, renal function, liver disease and nephrometry scores, were not consistently available across participating centres and therefore could not be included in the analyses. Organ volumes may also be influenced by unmeasured factors, including metabolic conditions, medication use, hydration status and other physiological factors. Second, differences in CT scanners, acquisition protocols and reconstruction parameters across centres may have introduced measurement heterogeneity despite standardized image preprocessing. Although study-specific validation demonstrated good agreement between automated and manual segmentation, some segmentation uncertainty remains, particularly for small structures such as the adrenal glands. Third, multiple organ measures and survival endpoints were evaluated, although FDR correction was applied to reduce the risk of false-positive findings. In addition, the cut-off values used for Kaplan-Meier analyses were data-driven and derived from the same cohort, potentially overestimating between-group survival differences; therefore, these threshold-based findings require independent validation. Fourth, although centre-stratified Cox models were used to account for institutional differences, residual centre effects cannot be entirely excluded. Furthermore, all participating centres were located in China, which may limit the generalizability of our findings to other populations and healthcare settings. External validation in independent international cohorts with longer follow-up is therefore warranted. Finally, longitudinal CT imaging was unavailable, precluding the assessment of temporal changes in organ volumes, and the biological mechanisms underlying the observed associations remain to be elucidated.

In conclusion, larger liver and right adrenal gland volumes were independently associated with poorer OS in patients with ccRCC after adjustment for clinicopathological factors and centre effects, whereas no significant associations with RFS remained after multiple-testing correction. These findings require further validation in independent cohorts.

## Ethics and consent to participate declarations

The retrospective study followed the Declaration of Helsinki and was approved by the ethics committee of Wuhan Union Hospital (No. 2024-0361-01), Tianjin Medical University Cancer Institute & Hospital (No. bc20251107), First Hospital of Shanxi Medical University (No. KYLL-2025-068) and Jiangsu Province Hospital (No.2022-SR-408). Given the retrospective design of this study and the use of anonymized data, the requirement for written informed consent was waived by the institutional ethics committees of the participating centres.

## Supplementary Material

Supplemental Material.docx

## Data Availability

The data underlying this article will not be made publicly available because of the privacy of the study participants. The data that support the findings of this study are included in this article, and all other supporting data are available on request from the corresponding author.
