# Supplementary material for: Association of AI-derived abdominal organ volumetry with postoperative outcomes in clear cell renal cell carcinoma: a multicentre study
Source: Ann Med. 2026 Sep 10;58(1):2730502. doi: 10.1080/07853890.2026.2730502 (PMC13573536; doi:10.1080/07853890.2026.2730502)
Supplement: Supplementary file 1 [file IANN_A_2730502_SM5702.docx]

**Supplementary Material**

**Association of AI-Derived Abdominal Organ Volumetry with Postoperative Outcomes in Clear Cell Renal Cell Carcinoma: A Multicenter Study**

**Table of Contents**

[**Supplementary Figures	3**](#_Toc237734863)

[Figure S1. Distribution of organ volume measurements.	3](#_Toc237734864)

[Figure S2. Distribution of organ volume measurements across different centers.	4](#_Toc237734865)

[Figure S3. Validation of automated abdominal organ segmentation.	5](#_Toc237734866)

[Figure S4. Comparison of body surface area-normalized abdominal organ volumes between patients with ccRCC and healthy controls in the WHUH cohort after propensity score matching.	6](#_Toc237734867)

[Figure S5. Center-specific subgroup analyses of the associations between liver and right adrenal gland volume measures and overall survival in patients with ccRCC.	7](#_Toc237734868)

[**Supplementary Tables	8**](#_Toc237734869)

[Table S1. CT scan parameters of four cohorts.	8](#_Toc237734870)

[Table S2. Detailed definitions and measurement methods of abdominal organ volumes.	9](#_Toc237734871)

[Table S3. Performance evaluation of automated abdominal organ segmentation.	10](#_Toc237734872)

[Table S4. Demographic characteristics of the healthy control cohort and the ccRCC cohort before and after propensity score matching.	11](#_Toc237734873)

[Table S5. Demographic characteristics of the healthy control cohort and the ccRCC cohort from WHUH before and after propensity score matching.	12](#_Toc237734874)

[Table S6. Associations between abdominal organ volumes and recurrence-free survival in ccRCC patients.	13](#_Toc237734875)

[Table S7. Multivariable Fine-Gray competing-risk analysis for recurrence in patients with ccRCC.	14](#_Toc237734876)

[Table S8. Sensitivity analysis of associations between raw organ volumes and postoperative outcomes with additional adjustment for BSA.	15](#_Toc237734877)

[Table S9. Incremental prognostic value of organ volume metrics beyond established risk models for overall survival.	16](#_Toc237734878)

[Table S10. Restricted mean survival time differences in overall survival according to organ volume metrics.	17](#_Toc237734879)

# **Supplementary Figures**

# **Figure S1. Distribution of organ volume measurements.**

# **Figure S2. Distribution of organ volume measurements across different centers.**

# **Figure S3. Validation of automated abdominal organ segmentation.**

# **Figure S4. Comparison of body surface area-normalized abdominal organ volumes between patients with ccRCC and healthy controls in the WHUH cohort after propensity score matching.**

# **Figure S5. Center-specific subgroup analyses of the associations between liver and right adrenal gland volume measures and overall survival in patients with ccRCC.**

# **Supplementary Tables**

# **Table S1. CT scan parameters of four cohorts.**

|  | WHUH cohort | TMUCIH cohort | FHSX cohort | JSPH cohort |
| --- | --- | --- | --- | --- |
| CT scanners | Siemens, Philips, Toshiba, and GE | GE, Philips, Siemens, United Imaging, and Toshiba | GE, Siemens and Philips | Siemens, Toshiba, and GE |
| Slice thickness | 0.625-5 mm | 0.625-5 mm | 0.625-5 mm | 0.625-5 mm |
| Slice interval | 1-1.5 mm | 1-1.5 mm | 1-1.5 mm | 1-1.5 mm |
| Detector rows | 64, 128, 256-section | 64, 128, 256-section | 64, 128, 256-section | 128, 256-section |
| Tube current | Automatic tube-current | Automatic tube-current | Automatic tube-current | Automatic tube-current |
| Tube voltage | 70-140 kV | 120-140 kV | 120 kV | 120 kV |
| Matrix | 512×512 | 512×512 | 512×512 | 512×512 |
| Contrast agent concentration | 300-350 mgI/ml | 350 mgI/ml | 370 mgI/mL | 350 mgI/mL |
| Contrast agent dosage | 1.5 ml/kg | 1.5 ml/kg | 1.5-2.0 ml/kg | 1.5 ml/kg |
| Contrast injection rate | 2.0-3.0 ml/s | 2.5-3.0 ml/s | 3.0 ml/s | 2.5 ml/s |

Note: SV, spleen volume; LV, liver volume; PV, pancreas volume; LAGV, left adrenal gland volume; RAGV, right adrenal gland volume; 3D, three-dimensional; BSA, body surface area.

# **Table S2. Detailed definitions and measurement methods of abdominal organ volumes.**

| Abbreviation | Full name | Unit | Details |
| --- | --- | --- | --- |
| SV | spleen volume | mL | The script loads 3D voxel data and spatial information-containing affine matrices using the nibabel library. It derives the actual physical voxel dimensions from the affine matrix to compute the volume of an individual voxel. Binary masks are then generated based on predefined organ label values, and the number of voxels belonging to each target organ is counted. Finally, the organ volume is obtained by multiplying the voxel count by the volume of a single voxel. |
| LV | liver volume | mL |  |
| PV | pancreas volume | mL |  |
| LAGV | left adrenal gland volume | mL |  |
| RAGV | right adrenal gland volume | mL |  |
| SV_BSA_ | spleen volume / BSA | mL/m^2^ | The organ volume was divided by body surface area to yield the corresponding organ index. |
| LV_BSA_ | liver volume / BSA | mL/m^2^ |  |
| PV_BSA_ | pancreas volume / BSA | mL/m^2^ |  |
| LAGV_BSA_ | left adrenal gland volume / BSA | mL/m^2^ |  |
| RAGV_BSA_ | right adrenal gland volume / BSA | mL/m^2^ |  |

Note: SV, spleen volume; LV, liver volume; PV, pancreas volume; LAGV, left adrenal gland volume; RAGV, right adrenal gland volume; 3D, three-dimensional; BSA, body surface area.

# **Table S3. Performance evaluation of automated abdominal organ segmentation.**

| Metrics | Dice | ICC | Mean Absolute Percentage Error |
| --- | --- | --- | --- |
| SV | 0.963 | 0.987 (0.970-0.994) | 4.70% |
| LV | 0.966 | 0.973 (0.958-0.983) | 4.81% |
| PV | 0.897 | 0.952 (0.927-0.969) | 6.87% |
| LAGV | 0.852 | 0.944 (0.913-0.964) | 9.09% |
| RAGV | 0.848 | 0.938 (0.906-0.960) | 8.99% |

Note: Dice, dice similarity coefficient; ICC, intraclass correlation coefficient; SV, spleen volume; LV, liver volume; PV, pancreas volume; LAGV, left adrenal gland volume; RAGV, right adrenal gland volume.

# **Table S4. Demographic characteristics of the healthy control cohort and the ccRCC cohort before and after propensity score matching.**

| Characteristics | Before PSM | | After PSM | |
| --- | --- | --- | --- | --- |
|  | ccRCC cohort  (n = 1720) | control cohort  (n = 3052) | ccRCC cohort  (n = 1465) | control cohort  (n = 1465) |
| Age | 57.0 (51.0, 65.0) | 44.0 (35.8, 54.0) | 56.0 (49.0, 62.0) | 55.0 (49.0, 60.0) |
| Gender |  |  |  |  |
| Male | 1122 (65.2%) | 1982 (64.9%) | 930 (63.5%) | 989 (67.5%) |
| Female | 598 (34.8%) | 1070 (35.1%) | 535 (36.5%) | 476 (32.5%) |

Note: ccRCC, clear cell renal cell carcinoma; PSM, propensity score matching.

# **Table S5. Demographic characteristics of the healthy control cohort and the ccRCC cohort from WHUH before and after propensity score matching.**

| Characteristics | Before PSM | | After PSM | |
| --- | --- | --- | --- | --- |
|  | ccRCC cohort  (n = 541) | control cohort  (n = 3052) | ccRCC cohort  (n = 540) | control cohort  (n = 540) |
| Age | 56.0 (49.0, 64.0) | 44.0 (35.8, 54.0) | 56.0 (49.0, 64.0) | 56.0 (49.0, 64.0) |
| Gender |  |  |  |  |
| Male | 354 (65.4%) | 1982 (64.9%) | 353 (65.4%) | 354 (65.6%) |
| Female | 187 (34.6%) | 1070 (35.1%) | 187 (34.6%) | 186 (34.4%) |

Note: ccRCC, clear cell renal cell carcinoma; WHUH, Wuhan Union Hospital; PSM, propensity score matching.

# **Table S6. Associations between abdominal organ volumes and recurrence-free survival in ccRCC patients.**

| Metrics | Univariate | | | Multivariate | | |
| --- | --- | --- | --- | --- | --- | --- |
|  | HR (95% CI) | *P* value | *P* adj | HR (95% CI) ^*^ | *P* value | *P* adj |
| Spleen | | | | | | |
| SV | 1.11 (0.96, 1.27) | 0.152 | 0.277 | 1.05 (0.90, 1.24) | 0.526 | 0.757 |
| SV_BSA_ | 1.11 (0.96, 1.28) | 0.171 | 0.277 | 1.03 (0.89, 1.21) | 0.682 | 0.757 |
| Liver | | | | | | |
| LV | 1.19 (1.02, 1.38) | **0.023** | 0.196 | 1.21 (1.02, 1.43) | **0.030** | 0.304 |
| LV_BSA_ | 1.18 (1.01, 1.37) | **0.039** | 0.196 | 1.15 (0.98, 1.36) | 0.089 | 0.443 |
| Pancreas | | | | | | |
| PV | 0.98 (0.84, 1.15) | 0.806 | 0.806 | 1.02 (0.86, 1.22) | 0.815 | 0.815 |
| PV_BSA_ | 0.93 (0.79, 1.10) | 0.394 | 0.437 | 0.96 (0.82, 1.14) | 0.672 | 0.757 |
| Left adrenal gland | | | | | | |
| LAGV | 1.11 (0.95, 1.29) | 0.194 | 0.277 | 1.08 (0.92, 1.28) | 0.357 | 0.715 |
| LAGV_BSA_ | 1.09 (0.94, 1.27) | 0.275 | 0.343 | 1.05 (0.89, 1.23) | 0.582 | 0.757 |
| Right adrenal gland | | | | | | |
| RAGV | 1.15 (0.99, 1.33) | 0.062 | 0.204 | 1.11 (0.94, 1.31) | 0.207 | 0.689 |
| RAGV_BSA_ | 1.14 (0.98, 1.32) | 0.082 | 0.204 | 1.08 (0.92, 1.26) | 0.357 | 0.715 |

Note: ccRCC, clear cell renal cell carcinoma; BSA, body surface area; HR, hazard ratio; CI, confidence interval; SV, spleen volume; LV, liver volume; PV, pancreas volume; LAGV, left adrenal gland volume; RAGV, right adrenal gland volume.

Hazard ratios are expressed per 1 standard deviation increase for continuous variables.

^*^Adjusted for age, gender, tumor size, laterality, surgical approach, pT stage, pN stage, ISUP grade, and tumor necrosis, with the baseline hazard stratified by center.

# **Table S7. Multivariable Fine-Gray competing-risk analysis for recurrence in patients with ccRCC.**

| Metrics | sHR (95% CI) | *P* value | *P* adj |
| --- | --- | --- | --- |
| SV | 1.02 (0.87, 1.19) | 0.819 | 0.924 |
| SV_BSA_ | 1.00 (0.85, 1.16) | 0.969 | 0.969 |
| LV | 1.17 (0.98, 1.40) | 0.089 | 0.824 |
| LV_BSA_ | 1.12 (0.93, 1.33) | 0.228 | 0.824 |
| PV | 1.02 (0.85, 1.23) | 0.831 | 0.924 |
| PV_BSA_ | 0.96 (0.80, 1.15) | 0.674 | 0.924 |
| LAGV | 1.09 (0.92, 1.29) | 0.339 | 0.830 |
| LAGV_BSA_ | 1.05 (0.89, 1.23) | 0.573 | 0.924 |
| RAGV | 1.10 (0.93, 1.31) | 0.247 | 0.824 |
| RAGV_BSA_ | 1.07 (0.91, 1.26) | 0.415 | 0.830 |

Note: ccRCC, clear cell renal cell carcinoma; sHR, subdistribution hazard ratio; CI, confidence interval; BSA, body surface area; SV, spleen volume; LV, liver volume; PV, pancreas volume; LAGV, left adrenal gland volume; RAGV, right adrenal gland volume; *P* adj, adjusted P value.

# **Table S8. Sensitivity analysis of associations between raw organ volumes and postoperative outcomes with additional adjustment for BSA.**

| Metrics | RFS | | | OS | | |
| --- | --- | --- | --- | --- | --- | --- |
|  | HR (95% CI) * | *P* value | *P* adj | HR (95% CI) * | *P* value | *P* adj |
| SV | 1.04 (0.88, 1.22) | 0.669 | 0.837 | 1.04 (0.84, 1.29) | 0.727 | 0.727 |
| LV | 1.19 (0.99, 1.42) | 0.059 | 0.293 | 1.41 (1.11, 1.79) | 0.004 | 0.022 |
| PV | 0.99 (0.83, 1.19) | 0.933 | 0.933 | 0.94 (0.73, 1.21) | 0.630 | 0.727 |
| LAGV | 1.09 (0.92, 1.29) | 0.316 | 0.791 | 1.34 (1.07, 1.67) | 0.009 | 0.023 |
| RAGV | 1.06 (0.89, 1.26) | 0.522 | 0.837 | 1.19 (0.95, 1.49) | 0.139 | 0.231 |

Note: ccRCC, clear cell renal cell carcinoma; HR, hazard ratio; CI, confidence interval; BSA, body surface area; SV, spleen volume; LV, liver volume; PV, pancreas volume; LAGV, left adrenal gland volume; RAGV, right adrenal gland volume; *P* adj, adjusted P value.

# **Table S9. Incremental prognostic value of organ volume metrics beyond established risk models for overall survival.**

| Model | C-index (95% CI) | *P* value | NRI (95% CI) | *P* value | IDI (95% CI) | *P* value |
| --- | --- | --- | --- | --- | --- | --- |
| pTNM | 0.6476 (0.5878, 0.7107) | Ref. | Ref. | Ref. | Ref. | Ref. |
| + LV | 0.6619 (0.5926, 0.7314) | 0.210 | -0.0286 (-0.2537, 0.2344) | 0.410 | 0.0022 (-0.0023, 0.0072) | 0.184 |
| + LV_BSA_ | 0.6645 (0.5925, 0.7325) | 0.161 | 0.0389 (-0.2157, 0.3021) | 0.389 | 0.0028 (-0.0017, 0.0074) | 0.116 |
| + RAGV | 0.6826 (0.6177, 0.7474) | **0.029** | 0.3975 (0.1710, 0.6433) | **0.001** | 0.0100 (0.0018, 0.0197) | **0.012** |
| + RAGV_BSA_ | 0.6831 (0.6191, 0.7479) | **0.026** | 0.4140 (0.1987, 0.6453) | **0.002** | 0.0115 (0.0029, 0.0218) | **0.003** |
| Leibovich score | 0.7076 (0.6390, 0.7709) | Ref. | Ref. | Ref. | Ref. | Ref. |
| + LV | 0.7139 (0.6470, 0.7787) | 0.211 | -0.0276 (-0.2556, 0.2374) | 0.422 | 0.0034 (-0.0019, 0.0093) | 0.120 |
| + LV_BSA_ | 0.7163 (0.6532, 0.7775) | 0.111 | 0.0342 (-0.2240, 0.2901) | 0.402 | 0.0034 (-0.0017, 0.0088) | 0.099 |
| + RAGV | 0.7221 (0.6593, 0.7819) | 0.073 | 0.2743 (0.0545, 0.5154) | **0.012** | 0.0115 (0.0029, 0.0223) | **0.005** |
| + RAGV_BSA_ | 0.7239 (0.6640, 0.7818) | 0.067 | 0.4091 (0.1935, 0.6368) | **0.002** | 0.0132 (0.0040, 0.0244) | **0.001** |

Note: pTNM, pathological tumor-node-metastasis staging; BSA, body surface area; LV, liver volume; RAGV, right adrenal gland volume; C-index, concordance index; CI, confidence interval; NRI, net reclassification improvement; IDI, integrated discrimination improvement; Ref., reference.

# **Table S10. Restricted mean survival time differences in overall survival according to organ volume metrics.**

| Metric | Low group RMST  (95% CI) | High group RMST  (95% CI) | RMST difference^*^  (95% CI) | *P* value |
| --- | --- | --- | --- | --- |
| LV | 69.65 (69.07, 70.22) | 68.44 (66.98, 69.89) | 1.21 (−0.36, 2.78) | 0.130 |
| LV_BSA_ | 70.25 (69.48, 71.02) | 68.98 (68.26, 69.70) | 1.27 (0.21, 2.32) | **0.018** |
| RAGV | 70.34 (69.69, 70.99) | 68.70 (67.88, 69.52) | 1.64 (0.60, 2.68) | **0.002** |
| RAGV_BSA_ | 70.18 (69.61, 70.74) | 67.95 (66.80, 69.09) | 2.23 (0.95, 3.51) | **<0.001** |

Note: RMST, restricted mean survival time; CI, confidence interval; LV, liver volume; RAGV, right adrenal gland volume; BSA, body surface area.

*RMST difference was calculated as Low group minus High group. RMST was estimated up to 72 months, when >10% of patients remained at risk.
